# Exome Study of Single Nucleotide Variations in Patients with Syndromic and Non-Syndromic Autism Reveals Potential Candidate Genes for Diagnostics and Novel Single Nucleotide Variants

**DOI:** 10.3390/cells14120915

**Published:** 2025-06-17

**Authors:** Lyudmila Belenska-Todorova, Milen Zamfirov, Tihomir Todorov, Slavena Atemin, Mila Sleptsova, Zornitsa Pavlova, Tanya Kadiyska, Ales Maver, Borut Peterlin, Albena Todorova

**Affiliations:** 1 Department of Biology, Medical Genetics and Microbiology, Faculty of Medicine, Sofia University “St. Kliment Ohridski”, 1000 Sofia, Bulgaria; 2 Center with an Autism Research Laboratory, Faculty of Educational Studies and Arts, Sofia University “St. Kliment Ohridski”, 1000 Sofia, Bulgaria; m.zamfirov@fppse.uni-sofia.bg; 3 Department of Special Pedagogy and Speech Therapy, Faculty of Educational Sciences and Arts, Sofia University “St. Kliment Ohridski”, 1000 Sofia, Bulgaria; 4 Genetic Medico-Diagnostic Laboratory Genica, 1000 Sofia, Bulgariakadiyska_t@yahoo.com (T.K.); todorova_albena@abv.bg (A.T.); 5 Independent Medico-Diagnostic Laboratory Genome Center “Bulgaria”, 1000 Sofia, Bulgaria; 6 Department of Physiology and Pathophysiology, Medical University of Sofia, 1000 Sofia, Bulgaria; 7 Clinical Institute of Genomic Medicine, University Medical Centre Ljubljana, 1000 Ljubljana, Sloveniaborut.peterlin@kclj.si (B.P.); 8 Department of Medical Chemistry and Biochemistry, Medical University of Sofia, 1000 Sofia, Bulgaria

**Keywords:** autism spectrum disorder, whole exome sequencing, single nucleotide variations, neuron structure, neuron function

## Abstract

Autism spectrum disorder (ASD) is a neurodevelopmental impairment that occurs due to mutations related to the formation of the nervous system, combined with the impact of various epigenetic and environmental factors. This necessitates the identification of the genetic variations involved in ASD pathogenesis. We performed whole exome sequencing (WES) in a cohort of 22 Bulgarian male and female individuals showing ASD features alongside segregation analyses of their families. A targeted panel of genes was chosen and analyzed for each case, based on a detailed examination of clinical data. Gene analyses revealed that specific variants concern key neurobiological processes involving neuronal architecture, development, and function. These variants occur in a number of genes, including *SHANK3*, *DLG3*, *NALCN*, and *PACS2* which are critical for synaptic signaling imbalance, *CEP120* and *BBS5* for ciliopathies, *SPTAN1* for spectrins structure, *SPATA5*, *TRAK1*, and *VPS13B* for neuronal organelles trafficking and integrity, *TAF6*, *SMARCB1*, *DDX3X*, *MECP2*, and *SETD1A* for gene expression, *CDK13* for cell cycle control, *ALDH5A1, DPYD*, *FH*, and *PDHX* for mitochondrial function, and *PQBP1*, *HUWE1*, and WDR45 for neuron homeostasis. Novel single nucleotide variants in the *SPATA5*, *CEP120*, *BBS5, SETD1A*, *TRAK1*, *VPS13B*, and *DDX3X* genes have been identified and proposed for use in ASD diagnostics. Our data contribute to a better understanding of the complex neurobiological features of autism and are applicable in the diagnosis and development of personalized therapeutic approaches.

## 1. Introduction

Autism spectrum disorder (ASD) is a neurodevelopmental impairment with early onset, characterized by social difficulties, deterioration of communication, repetitive behaviors, stereotyped and restricted interests, and atypical sensory stimuli perception [1,2,3,4]. Comorbid psychiatric and behavioral disorders are common in individuals with ASD, including ADHD, mood disorders, anxiety, obsessive compulsive disorder, irritability, aggression, substance use disorders, self-injurious behaviors, gender dysphoria, suicidality, psychosis, catatonia, and schizophrenia spectrum disorders [4]. Currently, the diagnostics of ASD are symptomatic and according to the *Diagnostic and Statistical Manual of Mental Disorders*-5th edition (DSM-5), it belongs to the group of neurodevelopmental disorders (NDDs) [5]. In the present medical nomenclature, ASD covers several NDDs: autism, atypical autism, Asperger’s syndrome, and a pervasive developmental disorder not otherwise specified.

The DSM-5 redefines ASD as a single continuum rather than a collection of distinct categories, which triggers a significant evolvement of the diagnostic criteria for ASD. The heterogenous nature of the spectra could be grouped into two critical domains: social communication deterioration and restricted, repetitive behaviors, that must present early in development and significantly affect daily functioning [6]. The Classification of Functioning, Disability, and Health for Children and Youth (ICF-CY) provides basic indicators which can be applied to different forms of ASD diagnosis (https://icd.who.int/en/ (accessed on 5 May 2025)). ASD has a large etiological heterogeneity due to variability in its phenotype observed in symptoms, onset, and severity. In these complex conditions, multiple genetic, epigenetic, and environmental factors additively contribute to symptom expression [7]. About 85% of the cases of ASD are estimated to have idiopathic ASD (with undefined causes for its pathogenesis), and just about 15% are considered to be secondary ASD (where a specific cause can be identified) [7]. No biomarkers are currently available for diagnosis or monitoring of ASD progression [8].

Molecular research has defined multigene dysfunction as an important causative factor of ASD, whereas its heterogeneity could be explained by the multigene model influenced by metabolomic factors [8]. Genetic studies have confirmed that ASD has a strong genetic basis and genetic heterogeneity [9]. It can be a distinct clinical phenotype or syndromic, related to the most common genetic syndromes [10]. Non-syndromic ASD could be polygenic and multifactorial, determined by specific combinations of environmental and genetic factors, or a single gene mutation can result in developing the disease in the relatively benign form of sporadic non-syndromic ASD [11]. In syndromic cases, ASD is a symptom of a more profound developmental disorder that includes multiple phenotypes, such as dysmorphic features, intellectual disability, and epilepsy [12]. The most common related with autism genetic syndromes comprise: Down, Fragile X, tuberous sclerosis complex, neurofibromatosis 1, Cornelia de Lange, Angelman, Coffin–Lowry, Cohen Laurence–Moon–Biedel, Marinesco-Sjogren, Moebius, Prader–Willi Rett syndrome, phenylketonuria, mitochondrial disorders, and many others [10,13]. The prevalence of ASD within these syndromes varies but can reach or exceed 60% [13]. The specific factors that determine the characteristics of subgroups of autism patients in each individual syndrome remain unknown [10].

Recent large-scale genomic studies using whole exome sequencing (WES) and chromosomal microarray analysis have significantly advanced our understanding of genetic contributions to ASD and related neurodevelopmental disorders [14]. These studies have identified novel genes with potential diagnostic value and suggest that clustering missense mutations indicates that normal development may be disrupted through activating or dominant-negative mechanisms [14]. Various genetic changes have been found in individuals with autism spectrum disorder (ASD), including missense and nonsense mutations, which alter protein function, and copy number variations, where segments of DNA are either duplicated or deleted, affecting gene expression [15]. Three major categories of genetic risk are implicated in ASD: common polygenic variations, rare inherited, and de novo mutations. Single nucleotide variants (SNVs), defined as rare nucleotide changes with an allele frequency < 1%, are of particular interest due to their potential to affect protein structure and function [16]. Pathogenicity of SNVsis evaluated according to guidelines from the American College of Medical Genetics and Genomics (ACMG) and the Association for Molecular Pathology (AMP), which classify variants as pathogenic, likely pathogenic, of uncertain significance (VUS), likely benign, or benign [17]. Dynamically growing data points to ASD association with a variety of genes and their single nucleotide variants (SNVs) [18]. A study based on the Australian database for ASD patients reports that single nucleotide variants (SNVs) are considered to account for 40–50% of ASD cases [19]. Lots of ASD candidate genes have been highlighted using genomic analyses for determining allelic diversity, mode of inheritance, and phenotypic impact of inherited and de novo variants of ASD and NDD genes [20,21,22,23,24,25,26]. According to the Simons Foundation Autism Research Initiative (SFARI) gene database, over 1000 gene candidates have been associated with ASD (About the Gene Scoring Module, https://gene.sfari.org/about-gene-scoring/ (accessed on 8 June 2025)). Mutations associated with autism often affect genes, related to a variety of metabolic disorders in the patients. Specific metabolites and metabolic pathways significantly differ in children with ASD compared to normally developing individuals [27].

SNVs may be either de novo arising during gametogenesis or embryonic development, or inherited from one or both parents [18]. It has been reported that postzygotic mutations account for numerous de novo harmful mutations resulting in mosaicism [28].

In this study, we performed whole exome sequencing on a cohort of 22 Bulgarian male and female individuals showing ASD features alongside segregation analyses of their families. We applied a targeted gene panel approach, selecting genes based on their clinical relevance to ASD and other neurodevelopmental conditions. This work aims to contribute to ASD gene discovery, support variant classification efforts, and improve understanding of the genetic architecture underlying ASD. Our goal was to identify rare de novo and inherited SNVs and assess their potential phenotypic impact, especially in relation to neurodevelopmental comorbidities.

## 2. Materials and Methods

In total, 22 Bulgarian patients (13 males and 9 females) with syndromic and non-syndromic autism spectrum disorder were selected from the medical records of Genetic Medico-Diagnostic Laboratory Genica, based on the joint consensus recommendation of the American College of Medical Genetics and Genomics and the Association for Molecular Pathology [17]. Each patient was clinically assessed by neurological and psychiatric examinations in children’s neurological and psychiatric clinics in Bulgaria. To assess structural and functional impairments in the brain, electroencephalography (EEG), computed tomography (CT), and magnetic resonance imaging (MRI) were used. Electromyography (EMG) was performed to evaluate muscle tone. The psychometric tests used for diagnosing ASD were Autism Diagnostic Interview Revised (ADI-R) [29], Autism Diagnostic Observation Schedule Generic (ADOS) [30], and Wechsler intelligence scale for children—5th edition. Some patients were diagnosed on the basis of fifth edition of *Diagnostic and Statistical Manual of Mental Disorders* (DSM-5) [5]. Written informed consent was obtained from the patients’ guardians, as well as from all family members tested.

High molecular weight DNA was extracted from EDTA-venous blood by standard salting-out method. The quality of the extracted DNA was assessed by direct spectrophotometry.

Whole exome sequencing (WES) was conducted in the partner laboratories “Admera Health, LLC, NJ, USA”, and Clinical Institute of Medical Genetics, UMC Ljubljana, Ljubljana, Slovenia. Clinically relevant genes were analyzed via a specialized software, GensearchNGS (PhenoSystems SA, Basel, Switzerland).

To verify the variants detected by WES, the target regions of the human genome, were multiplied by polymerase chain reaction (PCR). The amplified fragments were sequenced by Sanger’s method with BigDye^®^ Terminator v.3.1 sequencing kit (Applied Biosystems, Foster City, CA, USA). Electrophoretic separation of sequence products was performed via an ABI Prism 3130 Genetic Analyzer (Applied Biosystems, Foster City, CA, USA). The obtained data were processed automatically by the program ABI3130 Data Collection Software and received in the form of an electrophoregram with Sequencing Analysis software v.5.1.1. Additionally, segregation analysis in the family was performed via Sanger sequencing using parental DNA samples, extracted from venous blood. For the targeting sequencing, the list of utilized primers used to validate selected var-iants is provided in a Supplementary File (see Supplementary Table S1).

The interpretation of the detected genetic variants was performed according to the classification criteria of the guidelines of the American College of Medical Genetics and Genomics/Association of Molecular Pathology (ACMG/AMP), taking into account the clinical manifestations and the results from the segregation analyses performed in the families [17].

The study was conducted in accordance with the Declaration of Helsinki, and approved by the Ethics Committee of Sofia University “St. Kliment Ohridski”, Protocol No 93-M-412/1.10.2024 for studies involving humans (Approval date: 1 October 2024).

## 3. Results

The genetic variants which were detected by WES in our cohort of 22 Bulgarian patients (13 boys and 9 girls) having syndromic and non-syndromic phenotype are presented in Table 1. All patients share autistic features, neuropsychological delay, and intellectual disability. The detected genetic variants are classified as pathogenic, likely pathogenic, or VUS (variants of uncertain significance) according to the ACMG/AMP guidelines [17]. In total, 16 cases were concluded to carry pathogenic or likely pathogenic variants. In one of the cases, likely pathogenic and VUS were detected in the autosomal recessive *VPS13B* gene, which segregate in the family (one is maternally inherited and the other one is paternally inherited). Another patient carries a pathogenic de novo variant in the *SHANK3* gene and maternally inherited VUS in the *DLG3* gene. The last 4 cases carry variants classified as VUS in different genes (see Table 1). The performed segregation analysis in the affected families revealed 10 de novo cases, 11 cases with variants segregating in the family, and a case with one de novo and one maternally inherited variant. We identified novel single nucleotide variants in the *SPATA5*, *CEP120*, *BBS5*, *SETD1A*, *TRAK1*, *VPS13B*, and *DDX3X* genes, according to ClinVar (https://www.ncbi.nlm.nih.gov/clinvar (accessed on 5 May 2025)) and LOVD (https://www.lovd.nl/ (accessed on 5 May 2025)).

The cohort of 22 patients had been examined clinically and neuropsychologically in children’s neurological and psychiatric clinics in Bulgaria prior to genetic testing. All the patients share autistic features of poor social interaction, language use, speech deterioration, and repetitive behavior, combined with neuropsychological delay and intellectual disability. Their specific additional neurological and psychiatric profiles, as well as other clinical characteristics, accordingly with the findings from the WES analyses, are represented in Table 2.

## 4. Discussion

The underlying mechanisms of autism spectrum disorder (ASD) remain incompletely understood, with both inherited and de novo variants contributing to its complex etiology. Although ASD presents with a consistent behavioral profile, its causes are heterogeneous, involving genetic, chromosomal, and environmental components. Disruptions in genes regulating neuronal structure and function, even when not associated with well-defined syndromes, can lead to non-syndromic ASD.

We conducted whole exome sequencing (WES) and segregation analysis in 22 Bulgarian families with ASD probands and additional neurodevelopmental comorbidities. The cohort consisted of 13 males and 9 females (male-to-female ratio: 1.44), with a mean age at testing of 6.8 years (range: 2–34; SD: 7.01). The age of the patient varies due to the time when they were sent for WES. ASD appears more frequently in males than in females [31]. According to the statistics described by numerous authors, the male–female ratio is 4–5:1 [23]. Although ASD is more frequently diagnosed in males, the higher proportion of females in our cohort likely reflects the limited sample size and the tendency for genetic testing to be performed in cases with more severe phenotypes.

This study identified a range of potentially damaging de novo and inherited single nucleotide variants (SNVs), highlighting allelic diversity and helping to clarify inheritance patterns and genotype–phenotype correlations. Table 3 summarizes all detected SNVs, their functional roles in neurons, and SFARI gene classification where applicable.

Exome sequencing of 22 Bulgarian patients revealed SNVs affecting key intraneuronal mechanisms that align with each individual’s clinical presentation. These findings support the potential for personalized diagnostic and therapeutic approaches in autism and related neurodevelopmental disorders.

### 4.1. SNVs Associated with Synaptic Structure, Function, and Signaling Imbalance of Neurons in ASD

In our exome sequencing study of individuals with ASD, we identified rare variants in genes implicated in synaptic regulation and neuronal excitability, including *NALCN, PACS2, SHANK3,* and *DLG3*. These findings support the role of disrupted excitatory-inhibitory signaling in ASD pathogenesis. A de novo heterozygous missense variant in *NALCN* was detected in a female patient presenting with hypotonia, joint deformities, microcephaly, developmental delay, and ASD. The variant likely impairs sodium leak currents, contributing to neuromuscular dysfunction. A male patient with epilepsy, hypotonia, and ASD carried a de novo *PACS2* variant, potentially contributing to the ASD phenotype and the early-onset epileptic encephalopathy through impaired ion channel regulation. Another male patient with ASD had a de novo heterozygous splice-site variant in *SHANK3*. The alteration likely affects alternative splicing, leading to production of variable variants of SHANK3 molecules and potentially disrupting synaptic function. Notably, this patient also carries a hemizygous maternally inherited *DLG3* variant, which is not currently listed in the SFARI database but is classified in ClinVar as likely pathogenic/variant of uncertain significance (https://www.ncbi.nlm.nih.gov/clinvar/variation/224095/ (accessed on 5 May 2025)), associated with X-linked intellectual disability 90. The combined presence of *SHANK3* and *DLG3* variants coincide with progressive loss of communication skills and worsening ASD symptoms. These cases underscore the genetic complexity of ASD and suggest that multiple rare SNVs presented in a single person may contribute to more severe phenotypes.

### 4.2. SNVs Implicated in Mitochondrial Dysfunction and ASD

Variants in mitochondrial metabolism in *ALDH5A1*, *DPYD*, *FH*, *PDHX*, and *SPATA5* were identified implicating mitochondrial dysfunction in a subset of ASD cases. Compound heterozygous variants in *ALDH5A1* were found in a female patient with ASD, epilepsy, hypotonia, developmental delay, and motor coordination deficits. Given its role in GABA metabolism, ALDH5A1 deficiency likely disrupts neurotransmitter balance, contributing to the clinical presentation. A homozygous splice-site variant in *DPYD* was identified in a male patient with microcephaly, severe developmental delay, hypotonia, seizures, and ASD, potentially leading to toxic metabolite accumulation. A female patient with microcephaly, developmental delay, and seizures carried a homozygous missense variant in *FH*. As *FH* encodes fumarate hydratase, essential for mitochondrial energy production, the variant likely impairs neuronal energy balance. A homozygous *PDHX* variant was found in a female patient with cerebral atrophy, severe intellectual disability, leukodystrophy, and ASD phenotype. The variant is expected to impair glucose metabolism in the brain, resulting in neurodegeneration. Although *FH* and *PDHX* are not currently listed in the SFARI Gene database, their involvement in mitochondrial energy pathways and the presence of ASD-related features suggest they merit further investigation as ASD candidate genes. Additionally, compound heterozygous variants in *SPATA5* were identified in a male patient with EEG abnormalities, delayed psychomotor development, speech delay, and stereotypies. *SPATA5* is essential for mitochondrial dynamics and ATP production in neurons. Disruption of its function may impair axonal growth and synaptic signaling, contributing to the observed neurodevelopmental phenotype. This case is described in detail in a separate study [89].

### 4.3. SNVs Associated with ASD and Defects in Gene Expression: Transcription Factors, Chromatin Remodeling, and Histone Methylation

We identified rare variants in genes involved in transcription regulation, chromatin remodeling, and histone methylation, including *MECP2*, *TAF6*, *SMARCB1*, *DDX3X*, and *SETD1A*. These variants are likely to contribute to ASD phenotypes through disrupted gene expression and neurodevelopmental processes. A male patient with a hemizygous nonsense variant in *MECP2* showed symptoms overlapping with West syndrome and ASD features. *MECP2* is a key transcription regulator in the brain [62], where both overexpression and underexpression can cause neurodevelopmental disorders [60]. Mutations in MECP2 are the primary cause of Rett syndrome (RTT), a condition mostly affecting females, although affected males often experience more severe symptoms and reduced survival [95]. Besides Rett Syndrome, *MECP2* mutations have been identified in individuals with classic autism suggesting that loss-of-function mutations of *MECP2* may contribute to ASD [96]. Our findings emphasize the need for further investigation into the diverse roles of MECP2 variants in neuronal dysfunction and neurodevelopment. While *MECP2* mutations are primarily linked to Rett syndrome and classic autism, this case highlights the phenotypic variability associated with partial loss-of-function.

A homozygous *TAF6* variant was identified in a female patient with intellectual disability, ASD, muscular hypotonia, and cerebellar hypoplasia likely impairing neuronal gene expression. A de novo missense variant in *SMARCB1* was found in a male with ASD, seizures, and facial dysmorphism. Though not in the SFARI database, it may disrupt synaptic development. A de novo heterozygous missense variant in *DDX3X* was detected in a female patient with facial dysmorphism and developmental delay. Given its established role in RNA metabolism and high-confidence association with ASD, the variant likely impairs neuronal proliferation and differentiation. Another male patient presented with muscular hypotonia, developmental delay, and atypical autism, and carried a de novo heterozygous frameshift variant in *SETD1A*. The gene encodes a component of a histone methyltransferase complex, and the variant likely disrupts transcriptional regulation during neurodevelopment.

### 4.4. SNVs Associated with ASD and Cell Cycle Regulation, Ciliopathies, and Spectrin Function

In our ASD cohort, we identified a de novo heterozygous missense variant in *CDK13* in a male patient with cognitive impairment, moderate intellectual disability, and ASD. Given *CDK13*’s role in transcriptional regulation and RNA splicing, the variant may impair neuronal differentiation and synapse formation, supporting its potential involvement in ASD-related neurodevelopment.

Variants associated with ciliopathies were also observed. A male patient with compound heterozygous missense variants in *CEP120* presented with ASD, speech delay, epilepsy, dolichocephaly, and hyperprolinemia type I. *CEP120* plays a role in centriole biogenesis and cerebellar development; its disruption may underlie the neurological phenotype. Another male patient carried biallelic variants in *BBS5*, presenting with polydactyly, developmental delay, and ASD. *BBS5* encodes a subunit of the BBSome, essential for cilia function and intracellular transport. Although *CEP120* and *BBS5* are not currently listed in the SFARI Gene database, their roles in neurodevelopment suggest they may be relevant to ASD pathogenesis.

We also identified a de novo heterozygous missense variant in *SPTAN1* in a male patient with seizures and ASD traits. *SPTAN1* encodes a neuronal spectrin that supports membrane stability and synaptic signaling. The variant may disrupt the spectrin-actin cytoskeleton, affecting ion channel organization and contributing to the observed phenotype.

### 4.5. SNVs Associated with ASD and Affecting Neuronal Organelle Trafficking and Homeostasis

We identified variants in *TRAK1* and *VPS13B* genes involved in intracellular trafficking and organelle maintenance—processes critical for neuronal function. A female patient carried a de novo heterozygous missense variant in *TRAK1*, a gene involved in mitochondrial transport along axons and dendrites. Although previously associated with neurodevelopmental disorders, *TRAK1* is not currently listed in the SFARI database. Given the ASD features in our patient, its role in ASD warrants further investigation. Another female patient presented with compound heterozygous variants (frameshift and missense) in *VPS13B*. She exhibited severe neurodevelopmental delay, speech impairment, stereotypies, cerebral palsy, and skeletal malformations. *VPS13B* is essential for Golgi integrity and vesicle trafficking, and its disruption likely contributed to the complex phenotype observed.

We also identified variants in genes regulating neuronal homeostasis. A male patient carried a maternally inherited hemizygous variant in *PQBP1*, presenting with moderate intellectual disability, behavioral stereotypies, and progressive neuropsychiatric decline. PQBP1 is involved in immune responses and proteostasis, and the variant likely impairs synaptic maintenance over time. A de novo heterozygous frameshift variant in *WDR45* was found in a female patient with epileptic encephalopathy, developmental delay, and ASD features. *WDR45* regulates autophagy, and the variant likely disrupted neuronal clearance of damaged components. Another male patient carried a de novo hemizygous missense variant in *HUWE1*, presenting with microcephaly, epilepsy, severe intellectual disability, and marked ASD traits. *HUWE1* encodes a ubiquitin ligase essential for cortical development and protein degradation, and its disruption may explain the severity of the phenotype. These findings highlight the role of proteostasis and organelle maintenance in ASD pathogenesis.

**Figure 1:**
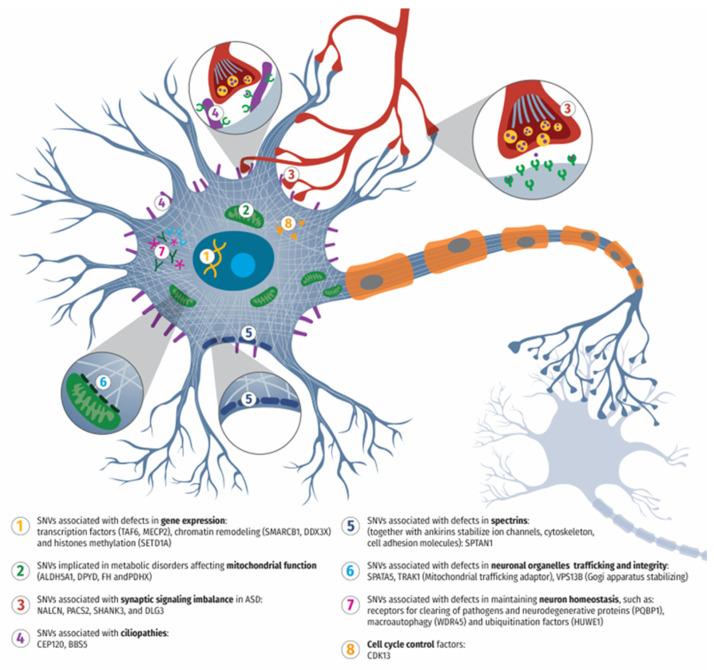
Schematic representation of neuronal structures affected by mutations identified in the cohort of 22 syndromic and non-syndromic patients exhibiting ASD-related phenotypic features.

The genes identified in our cohort are involved in critical neurobiological processes, including gene expression, mitochondrial function, synaptic signaling, organelle trafficking, neuronal homeostasis, cell cycle regulation, and the structural integrity of cilia and spectrins (Figure 1).

Among all the mutations mentioned, novel single nucleotide variants in the *SPATA5, CEP120*, *BBS5*, *SETD1A*, *TRAK1*, *VPS13B*, and *DDX3X* genes were identified in our cohort. These variants were not previously reported in the ClinVar (https://www.ncbi.nlm.nih.gov/clinvar (accessed on 5 May 2025)) and LOVD (https://www.lovd.nl/ (accessed on 5 May 2025)) databases. While associations with ASD have not been firmly established for genes like *SPATA5*, *CEP120*, and *TRAK1*, our findings suggest a potential role in ASD pathogenesis that warrants further investigation. Although *FH* and *PDHX* are not listed in SFARI, we detected homozygous variants in these genes in ASD patients, supporting their inclusion in future ASD-related metabolic gene panels.

There are several limitations and challenges to our study. ASD is highly heterogeneous, both genetically and clinically, necessitating large sample sizes for the discovery of rare genetic variations. Moreover, the demographic profile of the ASD patients in Bulgaria, together with the relatively small number of the Bulgarian population (about 6,718,470 people) and its economic status, results in the fact that only a small subset of the ASD patients have the opportunity to participate in genetic investigations. Genetic testing is primarily pursued by individuals presenting with severe psychosomatic and neurodevelopmental disorders, whereas those exhibiting milder or more limited ASD symptoms are often underrepresented or excluded from such diagnostic evaluations. Moreover, the lack of alignment of our data with findings reported in SFARI most probably is due to the fact that this database comprises results obtained by already performed investigations, but ASD is still not fully studied and unriddled.

## 5. Conclusions

Our findings support the view that autism spectrum disorders (ASDs) are genetically and clinically heterogeneous. Mutations implicated in ASD affect diverse neuronal functions, including synaptic architecture, excitatory-inhibitory balance, mitochondrial metabolism, and cellular processes such as chromatin remodeling, transcription regulation, organelle trafficking, and protein degradation. Several of these single nucleotide variants (SNVs) disrupt the epigenomic landscape, potentially contributing to the broad phenotypic variability observed in ASD.

Notably, SNVs in *SHANK3*, *DLG3*, and *PQBP1* may have prognostic value for neurodegenerative conditions with autistic features in later life. In our cohort of 22 patients, we identified novel SNVs in *SPATA5*, *CEP120*, *BBS5*, *SETD1A*, *TRAK1*, *VPS13B*, and *DDX3X* genes not currently listed in ClinVar or the SFARI database. These genes encode proteins essential for neuronal function and, given their presence in patients with ASD traits, merit further investigation as potential contributors to neurodevelopmental disorders.

In particular, SNVs in *SPATA5*, *CEP120*, and *TRAK1* may be linked to ASD-related symptoms in our patients.

Our study adds new data to public mutation databases and contributes to a more comprehensive understanding of autism’s molecular underpinnings, with implications for diagnosis and the development of targeted therapies.

## Figures and Tables

**Table 1:** Genetic variants detected by whole exome sequencing in a cohort of 22 Bulgarian patients. VUS—Variant of uncertain significance. * The classification is in accordance with the ACMG/AMP guidelines, Richards et al., 2015 [20].

Case	Gene ID	Sex	Age of Testing (Years)	Syndromic/Non-Syndromic Phenotype	Variant (GRCh37)	Variant Type	Zygosity	Inheritance	Pathogenicity *
1	*MECP2*	Male	2	Syndromic	chrX:g.153296071dup, NM_004992.3: c.1208dup, p.(*Glu404Ter*)	Nonsense	Hemizygous	Maternal	Likely pathogenic
2	*TAF6*	Female	15	Syndromic	chr7: g.99711522A>G, NM_001190415.1: c.323T>C, p.(*Ile108Thr*)	Missense	Homozygous	Biparental	Likely pathogenic
3	*SMARCB1*	Male	6	Syndromic	chr22: g.24145549C>T, NM_003073.3: c.568C>T, p.(*Arg190Trp*)	Missense	Heterozygous	de novo	Likely pathogenic
4	*PACS2*	Male	5	Syndromic	chr14: g.105834449G>A, NM_001100913.3: c.625G>A, p.(*Glu209Lys*)	Missense	Heterozygous	de novo	Pathogenic
5	*WDR45*	Female	4	Syndromic	chrX:g.48933330del, NM_007075.3:c.601_602del, p.(*Leu201LysfsTer21*)	Frameshift	Heterozygous	de novo	Likely pathogenic
6	*PQBP1*	Male	34	Syndromic	chrX:g.48760017C>T, NM_001032381.1:c.586C>T, p.(*Arg196Ter*)	Nonsense	Hemizygous	Maternal	Pathogenic
7	*SPATA5*	Male	13	Syndromic	chr4: g.123855300G>A, NM_145207.2: c. 554G>A, p.(*Gly185Glu*) p.	Missense	Heterozygous	Maternal	VUS
					chr4: g.123900503C>T, NM_145207.2: c.1831C>T, (*Pro611Ser*)	Missense	Heterozygous	Paternal	VUS
8	*NALCN*	Female	7	Syndromic	chr13: g.101944423A>G, NM_052867.2: c.965T>C, p.(*Ile322Thr*)	Missense	Heterozygous	de novo	Likely pathogenic
9	*FH*	Female	8	Syndromic	chr1: g.241667402G>A, NM_000143.4: c.1048C>T, p.(*Arg350Trp*)	Missense	Homozygous	Biparental	Likely pathogenic
10	*CEP120*	Male	3	Syndromic	chr5: g.122758670A>C, NM_153223.3: c.23T>G, p.(*Leu8Trp*)	Missense	Heterozygous	Paternal	VUS
					chr5: g.122700222G>C NM_153223.3: c.2548C>G, p.(*Arg850Gly*)	Missense	Heterozygous	Maternal	VUS
11	*BBS5*	Male	12	Syndromic	chr2:g.170343603G>A, NM_152384.3:c.167G>A, p.(*Arg56Lys*)	Missense	Heterozygous	Maternal	Likely pathogenic
					chr2:170354136G>C, NM_152384.3: c.619-1G>C	Splice site	Heterozygous	Paternal	Pathogenic
12	*SPTAN1*	Male	6	Syndromic	chr9: g.131394565C>T, NM_001130438.3: c.6922C>T, p.(*Arg2308Cys*)	Missense	Heterozygous	de novo	Likely pathogenic
13	*VPS13B*	Female	10	Syndromic	chr8:g.100844840_100844849delinsAC, NM_152564.5: c.9574_9583delinsAC, p.(*Val3192ThrfsTer33*)	Frameshift	Heterozygous	Paternal	Likely pathogenic
					chr8: g.100733139C>T, NM_152564.5: c.6914C>T, p.(*Thr2305Ile*)	Missense	Heterozygous	Maternal	VUS
14	*SHANK3*	Male	17	Non-syndromic	chr22: g.51153476G>A, NM_001372044.2: c.2490+1G>A	Splice site	Heterozygous	de novo	Pathogenic
	*DLG3*				chrX: g.69712394G>A, NM_021120.4:c.1721G>A, p.(*Arg574Gln*)	Missense	Hemizygous	Maternal	VUS
15	*CDK13*	Male	10	Syndromic	chr7: g.40085606A>T NM_003718.5: , c.2525A>T, p.(*Asn842Ile*)	Missense	Heterozygous	de novo	Pathogenic
16	*PDHX*	Female	3	Syndromic	chr11: g.35016549C>T, NM_003477.3: c.1336C>T, p.(*Arg446Ter*)	Nonsence	Homozygous	Biparental	Pathogenic
17	*SETD1A*	Male	15	Syndromic	chr16: g.30995020delG, NM_014712.3: c.4879del, p.(*Val1627TrpfsTer41*)	Frameshift	Heterozygous	de novo	Pathogenic
18	*TRAK1*	Female	5	Non-syndromic	chr3:g.42240742T>A, NM_001042646.3:c.1187T>A, p.(*Ile396Asn*)	Missense	Heterozygous	de novo	VUS
19	*ALDH5A1*	Female	3	Syndromic	chr6:g.24515433dup NM_170740.1:c.804dup, p.(*Val269fsTer19*)	Frameshift	Heterozygous	Paternal	Pathogenic
					chr6:g.24528277G>A NM_170740.1:c.1265G>A, p.(*Gly422Asp*)	Missense	Heterozygous	Maternal	Likely pathogenic
20	*DPYD*	Male	6	Syndromic	chr1:g.97915614C>T, NM_000110.3: c.1905+1G>A	Splice site	Homozygous	Biparental	Likely pathogenic
21	*DDX3X*	Female	2	Syndromic	chrX:g.41203374C>A, NM_001356.3:c.857C>A, p.(*Ala286Asp*)	Missense	Heterozygous	de novo	VUS
22	*HUWE1*	Male	8	Syndromic	chrX:g.53578038C>T, NM_031407.7:c.9209G>A, p.(*Arg3070His*)	Missense	Hemizygous	de novo	Pathogenic

**Table 2:** Neurological and psychiatric profiles, and the corresponding genetic analysis findings of the cohort of 22 Bulgarian patients.

Case	Gene ID	Sex	Age of Testing (Years)	Clinical and Neuropsychological Profile of the Patient
1	*MECP2*	Male	2	West syndrome: abnormal EEG, chaotic brain waves (hypsarrhythmia), specific infantile spasms with twitching of the head, arms, body tremors, stereotyped movements, and epileptic seizures, combined with axial muscular hypotony and motor developmental delay, mild fascial dysmorphism and smaller left auricle, clinodactyly of second left toe, and normal metabolic screening; communication deficits, lack of speech, and responding to commands, stereotyped movements.
2	*TAF6*	Female	15	Congenital cerebellar hypoplasia, hypotrophy (underrepresented subcutaneous fat tissue), ataxic gait, mild muscular hypotony, discretely impaired fine motor skills, mild mental retardation, defects in sound pronunciation, speech delay, mood disorders, insufficient concentration, and anxiety.
3	*SMARCB1*	Male	6	Epileptic seizures, focal epileptiform changes, and generalized paroxysmal manifestations of myocytic type, facial dysmorphism, delay in speech and neuropsychiatric development, deterioration in communication, infrequent eye contact, and stereotyped movements.
4	*PACS2*	Male	5	Microcephaly, facial dysmorphism, discrete facial symmetry, antimongoloid eye slits, epicanthus, hypertelorism, ocular coloboma, facial dysmorphism, backward rotated dysplastic auricles, muscular hypotony, epileptic seizures, and delay in speech and psychomotor development.
5	*WDR45*	Female	4	Epileptic encephalopathy with late epileptic spasms and focal seizures, moderate mental retardation, communication deficits.
6	*PQBP1*	Male	34	Confined atrophy of the brain, mental retardation since early childhood, behavioral stereotypes, tics, communication deficits.
7	*SPATA5*	Male	13	EEG abnormality, delayed onset of psychomotor development, speech delay, stereotypic movements.
8	*NALCN*	Female	7	Delay in speech, neuropsychiatric and psychomotor development, generalized muscular hypotension and hyporeflexia in the neonatal period, ulnar deviation of the fingers and hip dysplasia, speech and communication deterioration; lack of organic pathological changes in the examined intracranial anatomical components according to MRT of CNS and MR spectroscopy.
9	*FH*	Female	8	Microcephaly and unspecified encephalopathy; transfontanel ultrasound analyses shows mild diffuse dilatation of subarachnoid space and of the lateral ventricles; seizures, generalized muscular hypotension, delay in speech, and neuropsychiatric development.
10	*CEP120*	Male	3	Delay in speech and neuropsychiatric development, epileptic seizures, dolichocephaly, and hyperprolinemia type I.
11	*BBS5*	Male	12	Polydactyly, undeveloped expressive speech, communication deterioration, anxiety, psychomotor and sensory deterioration, and repetitive behavioral patterns.
12	*SPTAN1*	Male	6	Febrile seizures, behavior deterioration, and communication difficulties.
13	*VPS13B*	Female	10	Abnormal EEG, paralysis cerebralis, divergent strabismus, hydrocephaly, large fontanelle in infancy, planovagus deformity of the feet, pronounced scoliosis, severe neurodevelopmental disorder, speech delay, and stereotyped movements.
14	*SHANK3*	Male	17	Loss of previous skills over the years, loss of speech, and deterioration in communication.
	*DLG3*			
15	*CDK13*	Male	10	Moderate mental retardation, significant behavioral disorder requiring care and treatment; mental and physical developmental delay, lack of speech, stereotyped movements and behavior, lack of attention, and communication deficits.
16	*PDHX*	Female	3	Cerebral cortex atrophy, demyelination, mental retardation, significant behavioral disorder, alalia, central quadriplegia, microcephaly, blindness, and leukodystrophy.
17	*SETD1A*	Male	15	Neurobehavioral retardation, facial dysmorphism, muscular hypotony, enterocolitis, hepatosplenomegaly, and hemangioma parietis thoracis since birth.
18	*TRAK1*	Female	5	Speech delay and communication and behavioral deteriorations.
19	*ALDH5A1*	Female	3	Global developmental delay, including speech and behavioral disorders, hypotonia, coordination problems, hyporeflexia, movement disorders, and epilepsy.
20	*DPYD*	Male	6	Microcephaly, severe developmental delay, hypotonia, seizures, speech delay, and communication difficulties.
21	*DDX3X*	Female	2	Facial dysmorphism, delay in speech, and psychomotor development.
22	*HUWE1*	Male	8	Microcephaly, epilepsy, severe mental retardation, significant behavioral deterioration, lack of speech, and delay in motor development.

**Table 3:** Single nucleotide variants (SNVs) identified by whole exome sequencing in 22 Bulgarian patients. The table includes the affected gene and protein, a summary of each gene’s role in neuronal structure or function, symptoms observed in each of the 22 patients, and its classification according to the SFARI Gene database in relation to autism spectrum disorder (ASD).

Gene (SNV)	Protein Name	Role in Neuronal Structure or Function	SFARI Classification
NALCN	Sodium Leak Channel, Non-Selective	Regulates resting membrane potential and excitability [32,33,34]	Strong candidate
*PACS2*	Phosphofurin Acidic Cluster Sorting Protein 2	Synaptic signaling, organelle communication, calcium signaling, and mitochondrial function [35,36,37]	Syndromic
*SHANK3*	ProSAP SH3 and multiple ankyrin repeat domain protein 3	Synapse formation and maintenance [38,39,40,41,42]	High confidence
*DLG3*	disks large membrane-associated guanylate kinase scaffold protein 3, synapse-associated protein 102 (SAP-102)	Synaptic signaling involved in N-methyl-D-aspartate receptor clustering at excitatory synapses; synaptic plasticity [43,44]	Not listed
*ALDH5A1*	Aldehyde Dehydrogenase 5 Family Member A1	GABA metabolism, mitochondrial function [45,46,47,48,49]	High confidence, syndromic
*DPYD*	Dihydropyrimidine Dehydrogenase	Mitochondrial enzyme [50] and pyrimidine degradation [50,51,52,53]	Strong candidate
*FH*	Fumarate hydratase	Mitochondrial function, Krebs cycle enzyme; maintaining levels of neurotransmitters like glutamate, aspartate, and GABA [54]	Not listed
*PDHX*	pyruvate dehydrogenase X	Mitochondrial function; links glycolysis to tricarboxy acid cycle; neurotransmitter balance conversion of pyruvate to acetyl-CoA, maintaining levels of neurotransmitters like glutamate, aspartate, and GABA [42,55,56,57]	Not listed
*TAF6*	TATA-Box Binding Protein Associated Factor 6	Transcription initiation complex component [58,59]	Strong candidate
*MECP2*	Methyl-CpG Binding Protein 2	Transcription regulation [60,61,62]	High confidence, syndromic
*SMARCB1*	SWI/SNF-related, matrix- associated, actin-dependent regulator of chromatin subfamily B member 1	Chromatin remodeling complex subunit [63,64,65,66]	Not listed
*DDX3X*	DEAD-Box Helicase 3 X-Linked	RNA metabolism, translation initiation [67,68,69]	High confidence, syndromic
*SETD1A*	SET domain containing protein 1A or histone methyltransferase	Histone methylation and transcription regulation [70,71,72,73]	High confidence, syndromic
*CDK13*	cyclin-dependent kinase 13	Cell cycle control factors; transcriptional regulation and RNA splicing [74,75,76,77]	Syndromic
*CEP120*	centrosomal protein 120	Ciloigenesis, axonal growth, and cerebellar development [78,79]	Not listed
*BBS5*	BBSome	Cilia function and intracellular transport [80,81,82,83]	Not listed
*SPTAN1*	αII spectrin subunit	Membrane structure; synaptic support [84,85]	Not listed
*SPATA5*	spermatogenesis-associated protein 5	Mitochondrial dynamics; ATP production in neurons [86,87,88,89]	Not listed
*TRAK1*	trafficking kinesin binding protein 1	Mitochondrial transport in neurons; [90]	Not listed
*VPS13B*	vacuolar sorting protein 13	Golgi integrity; vesicle trafficking in neurons [91]	High confidence, strong candidate
*PQBP1*	Polyglutamine binding protein-1	Neuron homeostasis; clearance of neurotoxic proteins [92]	Not listed
*WDR45*	WD repeat-containing protein 45	Macroautophagy; removal of damaged organelles [93]	Not listed
*HUWE1*	HECT, UBA, and WWE Domain Containing E3 Ubiquitin Protein Ligase 1	Protein degradation and cortical development [94]	Syndromic

## Data Availability

Data are unavailable due to privacy and ethical restrictions.

## Supplementary Materials

The following supporting information can be downloaded at: https://www.mdpi.com/article/10.3390/cells14120915/s1. Supplementary Table S1. Primer sequences used for Sanger sequencing validation of rare and potentially pathogenic variants identified in ASD patients.
