# Supplementary material for: Exome Study of Single Nucleotide Variations in Patients with Syndromic and Non-Syndromic Autism Reveals Potential Candidate Genes for Diagnostics and Novel Single Nucleotide Variants
Source: Cells. 2025 Jun 17;14(12):915. doi: 10.3390/cells14120915 (PMC12191266; doi:10.3390/cells14120915)
Supplement: Supplementary file 1 [file cells-14-00915-s001.zip › cells-3604866-supplementary.pdf]

Gene (Verified Variant)	Primer Sequence
MECP2 (c.1208dup)	F - 5’-TGGTGAAGCCCCTGCTGGT-3’
	R - 5’-CTCCCTCCCCTCGGTGTTTG-3’
TAF6 (c.323T>C)	F - 5’-CGTCCTTAGGATTTTGGGCT-3’
	R - 5’-ACACACATCTGTCCTCCTTTCC-3’
SMARCB1 (c.568C>T)	F - 5’-CTCGCTGACTGTTGCTTCC-3’
	R - 5’-CAACCTCAGTATGGGGAAAG-3’
PACS2 (c.625G>A)	F - 5’-CTCTGTCCTGCATGGATCC-3’
	R - 5’-CCAAGACTCTCAGAGCCACC-3’
WDR45 (c.601_602del)	F - 5’-GTGGAGGGTTGAAGTCTGGT-3’
	R - 5’-CTGGCAATTTCCTCCAGGTTA-3’
PQBP1 (c.586C>T)	F - 5’-GGTGCTGTGGTACATGGCA-3’
	R - 5’-GTATCTTGCCGACTTGGGTT-3’
SPATA5 (c.554G>A)	F - 5’-CCAGATGGTGTTATAGGGGAATA-3’
	R - 5’-AGGGCTCTGTGTAACATCCAG-3’
SPATA5 (c.1831C>T)	F - 5’-CAGTGTCTTCACTGTTGCCACT-3’
	R - 5’-CCACTGGAGGAAACTGGGTATA-3’
NALCN (c.965T>C)	F - 5’-CCTGGCTTGAAGTCAGAAACA-3’
	R - 5’-TCCACTGATGGTGAGGGTG-3’
FH (c.1048C>T)	F - 5’-GGAACTTTCTGTTTCACTTGCTAAT-3’
	R - 5’-CCACAGACATGCTGGAACA-3’
CEP120 (c.23T>G)	F - 5’-CTTGAGTCGGGGCACAGT-3’
	R - 5’-CTCACTACGTACAACTTTGAACAAGTCT-3’
CEP120 (c.2548C>G)	F - 5’-CAGTTTTAGATGATGTCGTATGTTACACT-3’
	R - 5’-CAGTTTCTTCTGAGGAAAGGGA-3’
BBS5 (c.167G>A)	F - 5’-GGTTGGATATGCACAATATTTTCA-3’
	R - 5’-CTATATGTCCAAGACACAAAATGGA-3’
BBS5 (c.619-1G>C)	F - 5’-CTTTTACTTGATCTCTGTGGCAGTT-3’
	R - 5’-GATTCTTCCTCTGGGGACAA-3’
SPTAN1 (c.6922C>T)	F - 5’-GTTTTACGAGGTCTCAGGCCA-3’
	R - 5’-CTCACAGCACCTGGCCTTAA-3’
VPS13B (c.9574_9583delinsAC)	F - 5’-GAGGCATGAGAATTGCTTGAA-3’
	R - 5’-CACTTGACCCACTGTATACAATGTCTA-3’
VPS13B (c.6914C>T)	F - 5’-CATTCAATCTCTCTTAGGGGAAGTT-3’
	R - 5’-CCATGTATCATGAATTTGGATTACA-3’
SHANK3 (c.2490+1G>A)	F - 5’-CTGGAGTCTCCTTGAGGCTAGA-3’
	R - 5’-GCTGAGCTGTGGCCATTTA-3’
DLG3 (c.1721G>A)	F - 5’-GTGGTCTCCTGGAGACATCCT-3’
	R - 5’-GCAGTCAATGTTCTGATAGGACA-3’
CDK13 (c.2525A>T)	F - 5’-GATTAATTCTTCCTCTACCAGCCTT-3’
	R - 5’-CCTAATAGGTAACTTTCAACGCTCA-3’
PDHX (c.1336C>T)	F - 5’-GATTAAGGACATGCCTCCTTCA-3’
	R - 5’-CAACTAAGCTGAACACCAACTTCT-3’
SETD1A (c.4879del)	F - 5’-GAGCTGGAGGCAGAGCTG-3’
	R - 5’-CCAGTGGGAACTTGTAGTCGT-3’
TRAK1 (c.1187T>A)	F - 5’-CGTGAGCTTCTGATGCCTT-3’
	R - 5’-CTGGACTGGGAAGGACAATAGT-3’
ALDH5A1 (c.804dup)	F - 5’-CAGTTTGGTAAATTGTTGGCA-3’
	R - 5’-CCTGCGACAGAGCTTTTAACA-3’
ALDH5A1 (c.1265G>A)	F - 5’-CTCTGCAAATGTGGTTCCTTCTA-3’
	R - 5’-GCTCTTCAATGGACTCAACACA-3’

DPYD (c.1905+1G>A)	F - 5’-CTGCAAAAATGTGAGAAGGGA-3’
	R - 5’-CAAAGCAACTGGCAGATTCTTT-3’
DDX3X (c.857C>A)	F - 5’-GGGAATTATGTTGTGATGAACTTTT-3’
	R - 5’-AGTGGCTACTAACAAATGGCATC-3’
HUWE1 (c.9209G>A)	F - 5’-CTCAAGCAATTAATAAGGCGTGAAT-3’
	R - 5’-CCAGAGCCCTCCCACTTG-3’

Supplementary Table S1. Primer sequences used for Sanger sequencing validation of rare and potentially pathogenic variants identified in ASD patients. Each entry includes the gene name, variant notation (Human Genome Variation Society, HGVS), and forward (F) and reverse (R) primer sequences used for PCR amplification.
